# Retrospective analysis of 10,000 implants from insertion up to 20 years—analysis of implantations using augmentative procedures

**DOI:** 10.1186/s40729-016-0061-3

**Published:** 2016-12-03

**Authors:** Wolfram Knöfler, Thomas Barth, Reinhard Graul, Dietmar Krampe

**Affiliations:** 1 Praxis für Mund-, Kiefer-, Gesichtschirurgie und Implantologie, Rietschelstr. 27, 04177 Leipzig, Germany; 2 Dentale - Zahnärztliches Kompetenzzentrum GmbH, Prager Strasse 4, 04103 Leipzig, Germany; 3 Gemeinschaftspraxis für Mund-/Kiefer-/Gesichtschirurgie & Zahnheilkunde, Biedermannstr. 9-13, 04277 Leipzig, Germany; 4 DENTSPLY Implants, Steinzeugstraße 50, 68229 Mannheim, Germany

**Keywords:** Dental implant, Survival rate, Augmentation

## Abstract

**Background:**

A sufficient amount of bone is essential to ensure long-term stability of dental implants. To support the bone regeneration process, different techniques and materials are available. It has been questioned whether these techniques and materials may compromise implant survival compared to pristine bone. To properly answer this question, long-term stability up to 20.2 years after insertion of implants placed in augmented or non-augmented sites was retrospectively analysed.

**Methods:**

Retrospective analysis included 10,158 implants from 3095 patients in three private practices who underwent implant therapy with or without bone augmentation procedures. Different graft materials and membranes were used for augmentation. If necessary, the graft was stabilised using a titanium mesh. Implant survival was evaluated analysing explantation rates and Kaplan-Meier survival curves in augmented or non-augmented sites. In additional subgroup analyses, augmentation procedures, graft materials and membranes were compared applying descriptive statistics.

**Results:**

The observation period varied from the day of implantation up to 20.2 years after implant insertion. The overall implant survival was 95.5% (augmented sites 96.33%; native sites 94.27%). Comparison of Kaplan-Meier survival curves revealed significantly better survival of implants in augmented sites (*p* = 0.0025). When comparing different augmentation procedures, the best results were found for bone condensing followed by lateral augmentation. Graft materials were used in 58.2%, membranes in 36.6% of all implant sites. The most often used graft materials were a deproteinized bovine bone mineral (53.0%) and autogenous bone particles (32.5%). Both provided the best results and showed a significantly better implant survival compared to no graft material using the Kaplan-Meier method (*p* = 0.0104 and *p* < 0.0001). A native collagen membrane was used most often (74.0% of the membrane sites) and provided the best results regarding implant survival in the log-rank test.

**Conclusions:**

The retrospective analysis shows that implants inserted in augmented or native bone demonstrate similar implant survival under the conditions of private practice compared to prospective studies. To establish a broad base of support, further well-designed clinical trials are necessary.

## Background

Replacing missing teeth with dental implants is a routine treatment in many dental practices. In order to achieve adequate functional and aesthetic results, an optimal three-dimensional implant position has to be assured [1]. Various materials are available to build up missing bone. While autogenous bone is usually regarded to be the gold standard, harvesting may be associated with morbidity and considerable post-operative resorption of the augmented volume [2]. Therefore, bone substitutes are often used either alone or in combination with autogenous bone. Among bone substitutes, deproteinized bovine bone mineral has proven effectiveness in various indications as shown in clinical studies [3–7]. The long-term stability of the augmented volume found with this material is probably due to its slow resorption rate [8].

In guided bone regeneration procedures, membranes are often used to cover the graft and prevent ingrowth of soft tissue [9]. Native collagen membranes have been shown to allow bone formation with a low complication rate [6, 10–12].

Questions have been raised whether implant survival may be compromised in augmented sites since graft materials might impede and delay bone remodelling. While some studies reported reduced survival for implants in grafted areas [13, 14], other authors did not report any significant differences of implant survival or implant success between augmented and pristine bone [15, 16]. For sinus floor augmentation, Aghaloo et al. even found favourable implant survival rates in augmented bone [17].

Therefore, the objective of this study was to retrospectively analyse all consecutively placed implants in patients fulfilling the inclusion criteria and complete patient data files within 20 years in three private practices in terms of implant survival in augmented and non-augmented sites. Secondary objectives were to evaluate whether certain augmentation procedures or materials may be advantageous in terms of implant survival.

## Methods

The retrospective analysis evaluates patients who underwent implant therapy with or without accompanying augmentation procedures between August 1991 and December 2011 in three private practices. Strengthening the Reporting of Observational Studies in Epidemiology (STROBE) guidelines were followed. To investigate the effect of the different techniques applied on implant survival without overlapping impact of contraindications, the following exclusion criteria were applied: patients with uncontrolled diabetes mellitus, severe cardiovascular diseases (e.g. severe heart insufficiency), organ transplants, intake of bisphosphates and smoking of ≥20 cigarettes/day. Only patients with complete data regarding implantation procedure and implant survival were included. During this time period, a total of 10,165 implants were inserted either with or without augmentation. Seven implants were excluded from the evaluation because the date of implantation was not documented. Thus, 10,158 implants in 3095 consecutively treated patients were included in the retrospective analysis. Of these patients, 1693 (54.7%, 5626 implants) were female, 1401 (45.3%, 4539 implants) were male. For one patient, the sex was not documented. Mean age at the time of the implantation was 52.4 years (14.8 to 89.5). There was no difference regarding age or distribution of sex between patients with or without augmentation. On average, female patients received 3.32 and male patients 3.24 implants per patient.

Surgeries as well as pre- and postsurgical care were performed according to the standard procedures used in the three centres. Implants were inserted according to the manufacturers’ instructions. Patients were scheduled 3 months post-implantation followed by yearly control visits after the completion of the implant-supported restorative therapy.

The following graft materials were used: autogenous bone blocks, autogenous bone particles, Geistlich Bio-Oss (granules or collagen block, Geistlich Pharma AG, Wolhusen, Switzerland), Cerasorb (Curasan, Kleinostheim, Germany), Bioresorb (Implant Direct, Zurich, Switzerland), Bonitmatrix (DOT, Rostock, Germany), Biovin Bovine Bone (OT Medical, Bremen, Germany), Nanobone (Artoss, Rostock, Germany), Osteograf (Dentsply Tulsa Dental Specialities, Oklahoma, USA), Biogran (Biomet 3i, Munich, Germany), Easygraft (Degradable Solutions, Zurich, Switzerland), Endobone (Biomet 3i Deutschland GmbH, Munich, Germany), Pepgen P15 (Dentsply Tulsa Dental Specialities, Oklahoma, USA), Bioseed Oral Bone (Biotissue AG, Freiburg, Germany), Ostim (Heraeus Kulzer, Hanau, Germany), Perioglass (Novabone, Jacksonville, FL, USA) and Rebone (Schütz Dental GmbH, Rosbach, Germany). Autogenous bone was harvested during drilling and from the chin, tibial plateau, iliac crest, maxillary tuberosity and retromolar space.

The membranes applied included the native collagen membrane Geistlich Bio-Gide (Geistlich Pharma AG, Wolhusen, Switzerland) either alone or combined with one of the following membranes: Vicryl (Johnson & Johnson Medical GmbH, Norderstedt, Germany), Biovin Membran (OT Medical, Bremen, Germany), Parasorb Vlies (Resorba, Nuremberg, Germany), Gore-Tex Resolut (W.L. Gore & Associates, Flagstaff, USA), Kollagenresorb (RESORBA Medical GmbH, Nuremberg, Germany), Epi-Guide (DSM, Exton, USA), Gore Resolut Adapt Regenerative Membrane (W.L. Gore & Associates, Flagstaff, USA), Osseoguard (Biomet 3i, Munich, Germany), Ossix (Tel Aviv, Israel), Parasorb Resodont (RESORBA Medical GmbH, Nuremberg, Germany), Tefgen (Lifecore Biomedical, Chaska, USA), Tutodent (Tutogen, Neunkirchen, Germany), non-resorbable Gore-Tex membrane (GT, W.L. Gore & Associates, Flagstaff, USA), Osseoquest (W.L. Gore & Associates, Flagstaff, USA) and Inion GTR (Curasan, Kleinostheim, Germany). If a titanium mesh was used (Tiomesh, Dentaurum, Germany), the membranes were placed over the mesh.

Patient data files were analysed regarding personal patient information, implantation process and implantation outcome in terms of implant loss. Patient data included information about the sex, date of birth, the number and position of implants placed as well as the date of implantation and explantation or last control visit. Regarding the implantation procedure, the use of graft materials and membranes were documented.

Collected data were retrospectively analysed in terms of explantation rates to evaluate the survival between implants undergoing augmentation or not. Additional subgroup analysis included comparisons of different augmentation procedures, graft materials and membranes. In order to compare augmentation procedures, they were categorised into lateral augmentation, three-dimensional augmentation using a titanium mesh, bone splitting/bone spreading, use of autogenous bone blocks, internal sinus floor augmentation using the Osteotome technique and external sinus floor augmentation using a lateral window approach (one- and two-step procedure), bone condensing or combinations of these procedures. Bone splitting/spreading and bone condensing describe accompanying augmentation procedures to equalise the bone level with neighbouring sites.

### Statistical evaluation

The statistical analysis was performed using SPSS 11.0.0 (IBM, Armonk, NY, USA) as well as SAS Version 9.2 (SAS Institute Inc., Cary, NC, USA). Metric parameters were descriptively analysed for arithmetic mean and standard deviation. To test the hypothesis of “no differences between augmented sites and non-augmented sites in regard to survived implants”, survival of implants was compared based on Kaplan-Meier survival curves using log-rank test and included patient data up to a 20.2-year observation period [18]. Subgroups were exploratory analysed for statistical differences using log rank. A *p* value of 0.05 was regarded to be significant.

## Results

Of the 10,158 implants, 58.2% (5916 implants) were inserted using an augmentation procedure. The minimal observation period until the last control visit or until explantation was 0 days (day of implantation); the maximum period was 20.2 years. Distribution of analysed implants according to the period of observation is shown in Table 1.

**Table 1:** Distribution of implants according to the period of observation

Year	Number of implants	Relative number of implants (%)
<1	1920	18.9
1	1175	11.6
2	843	8.30
3	918	9.04
4	794	7.82
5	779	7.67
6	728	7.17
7	578	5.69
8	531	5.23
9	454	4.47
10	346	3.41
11	297	2.92
12	247	2.43
13	169	1.66
14	104	1.02
15	104	1.02
16	56	0.55
17	71	0.70
18	22	0.22
19	19	0.19
20	1	0.01
NA	2	0.02
Total	10158	100.0

*NA* observation period not clearly determinable

**Fig. 1:**
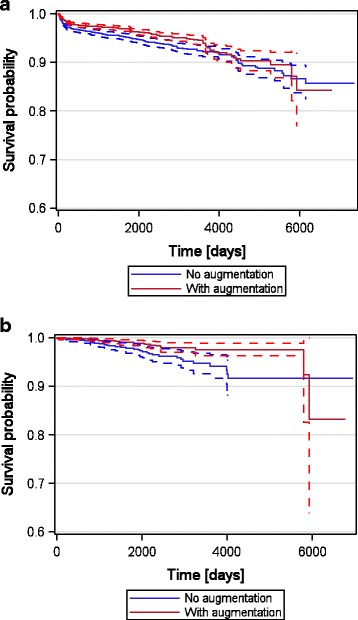
**a** Kaplan-Meier survival curves for implants with or without augmentation. *Dashed line* 95% confidence interval. **b** Kaplan-Meier survival curves for single crown implants overall and with or without augmentation. *Dashed line* 95% confidence interval

A total of 4.53% (460 implants) of all implants were lost during the observation period of 20.2 years. Analysis of early and late implant loss revealed that 16 implants (0.38%) without and 19 implants (0.32%) with augmentation were extracted before connection to the suprastructure, whereas 227 implants (5.35%) without and 198 implants (3.35%) with augmentation were lost after the attachment of the suprastructure within a 20.2-year observational period. Statistical analysis using Kaplan-Meier method and log-rank test revealed significantly better (*p* = 0.0025) survival curves for implants inserted with augmentation (96.33% of functional implants) compared to implants without augmentation (94.27% of functional implants) (Table 2 and Fig. 1). This was also true if only single-crown implants were evaluated, where the proportion of surviving implants was 98.84% (23 explantations of 1980 implants) with augmentation and 97.01% (34 explantations of 1136 implants) without augmentation (*p* = 0.0028).

**Table 2:** Implant loss in augmented and non-augmented sites up to 20.2 years after implant insertion

Augmentation	Implants (*n*)	Lost implants % (*n*)	Early implant loss % (*n*)	Late implant loss % (*n*)	Absolute survival rate %
No augmentation	4242	5.73 (243)	0.38 (16)	5.35 (227)	94.27
With augmentation	5916	3.67 (217)	0.32 (19)	3.35 (198)	96.33
Total	10158	4.53 (460)	0.34 (35)	4.18 (425)	95.47

Early implant loss (before connection of the suprastructure), late implant loss (after connection of the suprastructure). Metric parameters are calculated using descriptive statistics

### Augmentation procedures

**Fig. 2:**
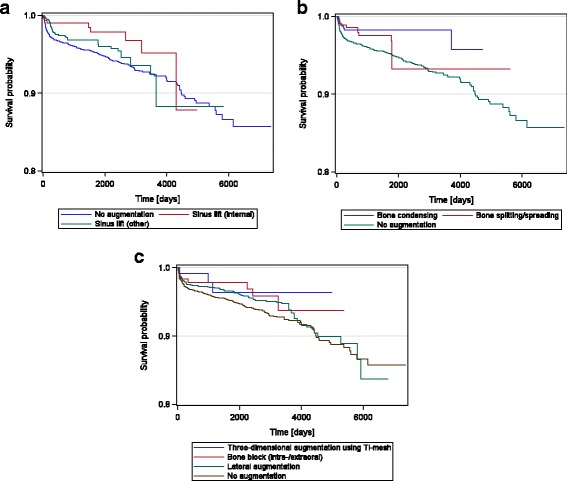
Kaplan-Meier implant survival curves for augmentation procedures

The augmentation procedures performed most frequently were lateral ridge augmentation and external one-step sinus floor augmentation (Table 3). During the observation period of 20.2 years, the percentage of surviving implants ranged between 95.0 and 98.5% and therefore slightly exceeded the one of no augmentation (Table 3). However, in pairwise comparisons, significant differences vs. no augmentation were only found for lateral augmentation as well as for external and internal sinus floor augmentations. In 57 implants, sinus lift was combined with other augmentation procedures. The absolute implant survival rate was 94.74% in these sites (*p* = 0.8826 vs. no augmentation). Kaplan-Meier implant survival curves are shown in Fig. 2.

**Table 3:** Explantations of implants inserted using different augmentation procedures up to 20.2 years after implantation

Augmentation procedure	Implants (*n*)	Lost implants % (*n*)	Absolute survival rate %	*p* value
No augmentation	4242	5.72 (243)	94.28	
Lateral augmentation	3210	4.02 (129)	95.98	0.0010
External sinus lift one-step	1101	4.09 (45)	95.91	0.0324
Bone condensing	422	1.90 (8)	98.1	0.0009
Bone splitting/bone spreading	374	3.74 (14)	96.26	0.2998
Internal sinus lift	314	2.55 (8)	97.45	0.0174
Autogenous bone block transplantation	241	3.32 (8)	96.68	0.1146
External sinus lift two-step	130	1.54 (2)	98.46	0.0410
Three-dimensional augmentation using Ti-mesh	124	2.42 (3)	97.58	0.1159

Metric parameters are based on descriptive statistics. *P* values for pairwise comparison vs. no augmentation were calculated in accordance to Kaplan and Meier using log-rank test.

When looking into the Kaplan-Meier implant survival curves of the augmentation procedures using the log-rank test, the highest implant survival was found for bone condensing followed by lateral augmentation, internal sinus lift, transplantation of bone blocks, bone splitting/spreading, titanium mesh, external sinus lift (one- and two-step) and finally, no augmentation. This sequence was statistically significant (*p* = 0.0336).

### Membranes

In 36.6% of all implant sites, a membrane was used. In 74.0% of these sites, the native collagen membrane Geistlich Bio-Gide was applied. Other often used membranes were Geistlich Bio-Gide combined with other membranes (7.38%), the non-resorbable Gore-Tex membrane (6.19%) and Vicryl (6.03%).

**Fig. 3:**
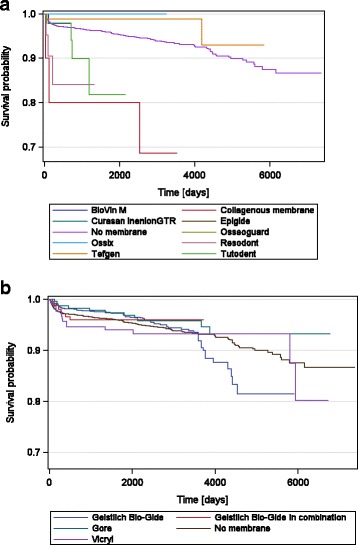
Kaplan-Meier survival curves for membrane types

In pairwise comparisons vs. no membrane, significantly increased rates of implant loss were found for Kollagen Vlies and Resodont, although the sample sizes were quite small (Table 4). When evaluating the Kaplan-Meier implant survival curves for the membranes (Fig. 3) using the log-rank test, the following sequence for implant survival was found (*p* = 0.0009): Geistlich Bio-Gide (highest survival), Gore-Tex, Tefgen, Ossix, Biovin, Ossoguard, Epigude, Inion, Geistlich Bio-Gide in combination with other membranes, Vicryl, Resodont, Kollagen Vlies, Tutodent, and no membrane (lowest survival).

**Table 4:** Explantations of implants per membrane type up to 20 years after implant insertion

Membrane type	Implants (*n*)	Min/max observation time (years)	Lost implants % (*n*)	Absolute survival rate %	*p* value
No membrane	6439	0.0/20.2	4.77 (307)	95.23	
Geistlich Bio-Gide	2743	0.0/16.2	3.76 (103)	96.24	0.4462
Geistlich Bio-Gide combined with other membranes	279	0.0/10.2	3.23 (9)	96.77	0.7809
Gore-Tex	230	0.4/18.5	5.65 (13)	94.35	0.1671
Vicryl	224	0.0/18.4	6.70 (15)	93.3	0.6808
Tefgen	81	0.2/16	2.47 (2)	97.53	0.1638
BioVin M	65	0/1.9	1.54 (1)	98.46	0.7816
Tutodent	49	0.1/5.9	8.16 (4)	91.84	0.0555
Resodont	21	0.1/3.6	14.29 (3)	85.71	0.0021
Kollagen Vlies	10	0.1/9.7	30.0 (3)	70	0.0006
Ossix	8	8.0/8.9	0.00 (0)	100	0.4781
Osseoguard	4	2.3/3.2	0.00 (0)	100	0.7061
Curasan InionGTR	3	1.0/1.0	0.00 (0)	100	0.7694
Epigide	2	4.9/4.9	0.00 (0)	100	0.7680
Total	10158	0.0/20.2	4.53 (460)	95.47	

Metric parameters were calculated using descriptive statistics. *P* values for pairwise comparison vs. no membrane were calculated in accordance to Kaplan and Meier using log-rank test

### Graft materials

The graft materials used most often were the deproteinized bovine bone mineral, Geistlich Bio-Oss (53.0%) and autogenous bone particles (32.5%). The use of both graft materials resulted in higher absolute implant survival assessing the data to no graft material (*p* = 0.0104 and *p* < 0.0001). In contrast, Cerasorb showed lower implant survival compared to no graft material (*p* = 0.0002). For the other materials, no differences were found (Table 5).

**Table 5:** Implants lost and in function up to 20.2 years after implant insertion using different graft materials

Graft material	Implants (*n*)	Min/max observation time (years)	Lost implants % (*n*)	Absolute survival rate %	*p* value
No graft material	4609	0.0/20.2	5.51 (254)	94.49	
Geistlich Bio-Oss	2939	0.0/15.6	2.76 (81)	97.24	0.0004
Autogenous bone particles	1801	0.0/17.8	3.94 (71)	96.06	0.1807
Cerasorb	284	0.0/12.6	10.56 (30)	89.44	0.0007
Bioresorb	145	0.1/11.6	6.90 (10)	93.10	0.7782
Bio-Oss + Cerasorb	105	0.0/5.8	2.86 (3)	97.14	0.6714
Other bone substitutes	275	0.0/18.6	4.00 (11)	96	0.1354
Total	10158	0.0/20.2	4.53 (460)	95.47	

Metric parameters were calculated applying descriptive statistics. *P* values for pairwise comparison vs. no graft material were calculated in accordance to Kaplan and Meier using log-rank test.

**Fig. 4:**
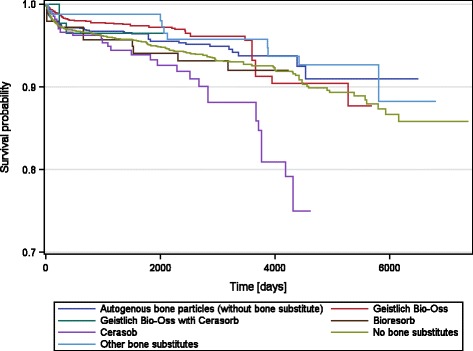
Kaplan-Meier implant survival curves for bone and bone substitutes

When comparing the Kaplan-Meier implant survival curves of the grafts (Fig. 4) to each other using the log-rank test, the following sequence for implant survival was found (*p* = 0.0001): Geistlich Bio-Oss (highest survival), other bone substitutes, autogenous bone particles, Geistlich Bio-Oss + Cerasorb, Bioresorb, Cerasorb and no graft material (lowest survival).

## Discussion

The retrospective analysis presented here evaluates implants inserted in three different private practices with or without augmentation procedures. Treatments were performed according to the standard protocols applied in the private practices. More than 10,000 implants were inserted in various indications and were followed up to 20.2 years from the day of implant insertion. The overall implant survival rate was 95.5%. When only single-crown implants were evaluated, the absolute survival rate increased to 98.8%. Various reviews have reported about the implant survival similar to the results found here [15, 17, 19].

In our analysis, survival of the 10,158 implants analysed was slightly but significantly higher in augmented bone than in pristine bone (96.3 vs. 94.3%). This might result from an increased mineral density, as usually observed after augmentation, and the concomitant higher bone-to-implant contact [20]. High number of implants analysed here allowed suited statistical analysis despite of patient- and implant-specific variations. According to the statistical results of survival curves, the hypothesis of no difference might be withdrawn in favour for augmented bone indicating a statistically significant positive effect of grafting on implant survival. However, small difference in absolute numbers should be carefully evaluated for clinical relevance. Previously, published studies regarding implant survival between augmented and non-augmented sites are inconclusive. In one retrospective analysis which included 12,737 implants in 4206 patients, 59.7% of the implants were inserted using bone augmentation or bone expanding procedures [16]. The authors did not find a significant difference between the Kaplan-Meier cumulative survival rates among grafted and non-grafted sites. In a recent review which included 108 articles of all evidence levels, Jensen and Terheyden found a high level of evidence that survival rates of implants in augmented bone are very similar to the ones of implants in pristine bone [15]. In another review, Aghaloo et al. reported similar or even better results for implants in augmented sites [17]. However, there are also a few clinical studies in which reduced survival rates for implants inserted in grafted areas were found [13, 14]. Differences in numbers of implants analysed, surgical techniques, indications and/or graft materials may account for these inconsistent results and further studies might be needed.

In the retrospective analysis shown here, the comparison of different augmentation procedures using the log-rank test revealed the highest implant survival for bone condensing followed by lateral ridge augmentation. The lowest rankings were found for sinus floor augmentation and no augmentation. In pairwise comparisons of Kaplan-Meier implant survival curves to non-augmented sites, a significantly higher implant survival was found for lateral bone augmentation and sinus floor elevation. However, all procedures provided a high implant survival of more than 94%. This indicates that under daily practice all these augmentation procedures may provide clinically acceptable results. Recent reviews have also reported a high implant survival of more than 90% for sinus augmentation [5, 15, 17, 21–23], lateral ridge augmentation [15, 19, 24, 25], for bone splitting [21, 26] as well as for three-dimensional augmentations using titanium mesh [27]. When augmentation procedures were compared to each other, the authors were not able to draw a clear conclusion on the superiority of a certain augmentation procedure or grafting protocol [15, 17, 21, 28].

In our analysis, membranes were used in 36.6% of the implant sites. Small defects were treated with either a bone substitute or bone particles without an additional membrane. The membrane which was used in almost 75% of the cases was a native collagen membrane. Various other studies have reported successful results using this membrane in bone augmentation [6, 10–12] as well as a low complication rate [29, 30]. Although in our analysis, the membrane was associated with a high absolute survival rate of 96.24% and the best result in the log-rank test, it is still not possible to draw clear conclusions on the superiority of any membrane, when implant survival is the only parameter under consideration.

The most often used graft material in our evaluation was Geistlich Bio-Oss (53.0%) followed by autogenous bone (32.5%). When compared to no graft, the use of both grafts resulted in significantly higher implant survival rates. In various studies, the bone substitute was found to promote bone regeneration and to allow for long-term stability of the augmented volume [3, 5–8, 31–33]. A recent meta-analysis compared Geistlich Bio-Oss and autogenous bone [24]: For maxillary sinus floor augmentation, a mean implant survival rate of 98.6 ± 2.6% was found for bone substitute, 88.6 ± 4.1% for autogenous bone + bone substitute and 97.4 ± 2.2% for autogenous bone alone. While there was a trend in favour of Geistlich Bio-Oss, the differences were not statistically significant. When the authors evaluated the studies with vertical and/or lateral alveolar ridge augmentation, they found similar mean implant survival rates for the three treatment modalities (97.4 ± 2.5% for Geistlich Bio-Oss, 100 ± 0.0% for autogenous bone + Geistlich Bio-Oss, 98.6 ± 2.9% for autogenous bone alone). Data is based on 4687 implants in 1816 patients. This finding is in line with the results from our study. In contrast to Geistlich Bio-Oss, the use of a synthetic material (Cerasorb) resulted in a significantly lower implant survival than no graft in the pairwise comparison, although in the log-rank test, the synthetic material demonstrated better results than no graft material used. However, the heterogeneity of the data does not allow drawing statistical conclusions on the superiority between those bone substitutes that were used rather rarely.

The retrospective analysis presented here included a large number of implants followed up to 20.2 years. While it allows conclusions on the efficacy of augmentation procedures in daily practice, there are some limitations. It was not possible to evaluate the initial defect size and morphology. Therefore, it is not clear whether the different graft materials and membranes were used in comparable clinical situations or whether differences in original defect size may have accounted for some of the differences in survival rates. In addition, the data were only evaluated for implant survival and not for implant success. This is due to the fact that in clinical studies, the success of dental implants is commonly defined by implant survival. Although different criteria for implant success were suggested in the 1980s and early 1990s [32, 34–37], the success rate addressing prosthetic, biological and aesthetic complications was largely absent from the literature in the 1990s and was newly established only 5 to 10 years ago. Despite of the international proposed criteria, a common consensus could not be reached so far. In addition, Buch et al. compared the different criteria proposed for implant success with regard to their clinical value [38]. The authors demonstrated that the proposed criteria led to very different success rates 6 years after implant insertion (75–89%) and did not allow reliable comparison of the results with each other. Thus, during control visits in our practices, only prosthetic complications, but no other factors essential on reporting implant success rates were documented. Especially in the anterior maxilla, the documentation of factors essential for a good aesthetic outcome with long-term stability would be of high importance. Another fact is that all inserted implants were intentionally included, irrespective of the indication, the augmentation materials used and irrespective of whether they were already in function or not. It would be of interest to evaluate the potential impact of different implantation and augmentation procedures on early and late implant loss as other authors could show remarkable effect on early and late implant failure [39, 40].

Due to this, clear conclusions on the advantages of certain procedures, materials, healing or loading times are not possible and might be subject to further discussion. According to a retrospective 10-year observation, most implant failures occurred before loading [41]. In most of the cases, the clinical cause was unclear, but 17.5% were due to iatrogenic conditions and only 3% could be attributed to poor bone quality and quantity. This, together with our own analysis suggests that early implant loss is related to a learning curve and the surgeons’ experience, as we have encountered that early implant loss was halved after approximately every 500 implants.

Nevertheless, our analysis indicates that under the conditions of daily practice, implants in augmented bone have survival rates that tend to be even better than implants inserted in native bone.

## Conclusions

In this retrospective analysis, more than 10,000 implants were included followed up to 20.2 years. They were inserted in a variety of indications either with or without augmentation procedures. While it was not possible to draw clear conclusions on the superiority of a certain augmentation procedure, a graft material or a membrane as the indication for the different materials and procedures might vary; the data indicated that implant survival in augmented bone may be slightly better than in pristine bone. Further well-designed, prospective, randomised, long-term studies are needed to get greater insights into this subject.
